# Microsatellite Markers for the Chameleon Grasshopper (*Kosciuscola tristis*) (Orthoptera: Acrididae), an Australian Alpine Specialist

**DOI:** 10.3390/ijms130912094

**Published:** 2012-09-24

**Authors:** Kate D. L. Umbers, Siobhan Dennison, Czarina A. Manahan, Laurence Blondin, Christine Pagés, Ange-Marie Risterucci, Marie-Pierre Chapuis

**Affiliations:** 1 Department of Biological Sciences, Macquarie University, Sydney, NSW 2109, Australia; E-Mails: siobhan.dennison@mq.edu.au (S.D.); czarina_112@hotmail.com (C.A.M.); 2 CIRAD, UPR Bioagresseurs analyse et maîtrise du risque, F-34398 Montpellier, France; E-Mails: laurence.blondin@cirad.fr (L.B.); christine.pages@cirad.fr (C.P.); ange-marie.risterucci@cirad.fr (A.-M.R.); marie-pierrre.chapuis@cirad.fr (M.-P.C.)

**Keywords:** Australia, alpine, grasshopper, colour, color, thermoregulation, sexual selection, population genetics, conservation genetics, climate change, male competition

## Abstract

A set of polymorphic loci was characterised using an enrichment library for the Australian alpine specialist, the chameleon grasshopper (*Kosciuscola tristis*), an atypical grasshopper known for its remarkable temperature-controlled colour change. The number of alleles per locus ranged from three to 20 and observed heterozygosity from 0.16 to 0.76. These are the first microsatellite markers for a non-endangered Australian alpine animal and will inform questions of gene flow across the sky islands of this unique and threatened region.

## 1. Introduction

The Australian alpine region has received little scientific attention despite its unique and threatened status [1]. Therefore, this large unspoilt stretch of wilderness harbours many new and little-known species. The *Kosciuscola* grasshoppers are endemic to the region with four mainland species (*Kosciuscola tristis*, *K. usitatus*, *K. cognatus*, *K. cuneatus*) and one in Tasmania (*K. tasmanicus*). Their altitudinal distribution ranges from 1000 m (in Tasmania) to the peak of Mt. Kosciuszko at 2228 m and from west of Canberra, 650 km south to just north of Melbourne, Victoria [2]. In Tasmania the distribution of *K. tasmanicus* spans 300 km along the north western alpine plateau. Though they are restricted to the comparatively small alpine region of Australia (just 0.04% of the continent), they are abundant during late summer and early autumn.

*Kosciuscola* grasshoppers are short-winged, flightless acridids (short-horned grasshoppers of which there are 8000 species). A most remarkable feature of this genus is that male *K. tristis* change colour in response to ambient temperature from black under 10 °C to turquoise above 25 °C [3,4]. This characteristic is not known in any other group of acridid. Given its distribution, *K. tristis* is an important research subject for understanding distribution patterns, population structure and reproductive isolation among patches of high altitude (“sky islands”) in the Australian Alps. This set of microsatellite markers is the first for an alpine invertebrate in Australia; so far only critically endangered vertebrate species with tiny populations have been genetically described because of immediate extinction risk [5,6]. This is also one of very few microsatellite sets for grasshoppers. Microsatellite data for *K. tristis* will highlight fine scale allelic distribution across their restricted range and provide greater insight into the processes important to the evolution of similarly isolated alpine species. Moreover, such data will provide a baseline from which the effects of climate change in the region can be measured as global temperatures rise.

## 2. Results and Discussion

Polymorphism and allelic distribution of eight loci were estimated for 26 *K. tristis* collected from one population located at Dead Horse Gap, Kosciuszko National Park, Australia using GenAlEx 6.2 [7] (Table 1). PCR products labelled with different coloured fluorescent tags using the M13 tagging method (see [8]) were pooled and visualised on a 3130xl Genetic Analyser (Applied Biosystems). Allele sizes were scored using Peak Scanner software (version 1.0 Applied Biosystems), and each genotype was also checked by eye. In the polymorphic loci the number of alleles ranged from four to twenty (Table 2).

No loci were in linkage disequilibrium but several of the loci deviated significantly from Hardy-Weinberg equilibrium (*p* < 0.01) (Genepop 3.4, [9]) (Table 2). All showed a homozygote excess evenly distributed across allele size classes, which excludes genotyping errors as the source of disequilibrium (Micro-Checker; [10]). Null alleles may have caused the observed deviation from Hardy-Weinberg equilibrium, with null allele frequencies estimated between 10% and 35% for K.tr29, K.tr30, K.tr76, K.tr82, K.tr88 (Table 2). A similar prevalence of null alleles is regularly observed in microsatellites of Orthoptera species [11–13]. Fortunately, methods are available to analyse microsatellite data in the presence of null alleles (e.g., FreeNA [12]). The deviation from Hardy-Weinberg equilibrium and reduced observed heterozygosity may be partially attributable to a Wahlund effect due to the isolated nature of the population and it is unclear for how long this population may have been isolated. The Dead Horse Gap population of *K. tristis* may have historically interbred with populations across the alpine plateau at Charlotte Pass (M. F. Day pers. comm.) but are now isolated from that population perhaps by shifts in distributions due to climatic change. Alternatively, if the population has been isolated for many generations, the excess of homozygotes observed in these data may represent inbreeding. Genotyping more populations will help assess whether the deviation from Hardy-Weinberg equilibrium is population-specific and shed light on gene flow and dispersal patterns within this region.

## 3. Experimental Section

A partial DNA library of recombinant clones was obtained from a single *K. tristis* individual originating from Dead Horse Gap, following the enrichment protocol from [15]. Total genomic DNA was extracted using salting-out [16] and digested using Rsa1 restriction enzyme. Digested DNA was ligated to oligo adapters Rsa21 and 5′-phosphorylated Rsa25, pre-amplified by polymerase chain reaction (PCR) using the adapter primers and purified (QIAquick PCR purification kit, QIAGEN). Biotinylated oligo probes I8(GA)_15_ and I8(GT)_15_ were hybridized to the ligated DNA and captured using streptavidin magnetic particles (Promega). The oligo adapter Rsa21 was used as primer to perform PCR on the microsatellite-enriched eluates. The amplification products were ligated into a plasmid vector (pGEM-T Easy, Promega) and transformed into ElectroMAX DH10B T1 resistant cells. Recombinant colonies were randomly selected and cloned inserts were amplified with the Rsa21 primer. The PCR products were transferred to nylon membranes (Hybond Inc) and probed with ^32^P-labelled sequences complementary to the microsatellites (CT)_15_ and (CA)_15_.

Screening of 288 cloned fragments identified 116 positive transformants (40%). Among them, 89 inserts were sequenced (performed by Cogenics). Forty-two of these sequences were excluded because of similar flanking sequences that were detected using the program Microfamily (*i.e.*, 47% [17]). High levels of similarity between flanking regions of microsatellite-containing sequences is often found in grasshoppers [11,18]. We selected 24 of the unique sequences, with an adequate flanking sequence size, for polymerase chain reaction (PCR) amplification.

Primers were designed for 24 sequences containing microsatellite repeats (Primer 3 Plus). PCRs were 10 μL reactions containing 50 ng–100 ng of genomic DNA, 200 μM of each dNTP, 2 μL of 5× GoTaq FlexiBuffer (Promega) and 0.02 μM fluoro-labelled tag, with locus-specific primer and MgCl_2_ concentrations (Table 2). The greatest resolution for all primer pairs was found with the following protocol: one initial denaturation at 94 °C for 3 min, then denaturation: 30 s at 94 °C, annealing: one cycle each at 60 °C, 58 °C, 56 °C, 54 °C and 52 °C, then 35 cycles at 50 °C for 30 s, extension: 72 °C for 45 s, and a final extension step of 72 °C for 10 min (MJ Research PTC100 Thermocycler). PCR product was visualised on 2% agarose gels with HyperLadder IV (BIOLINE) and eight primer pairs were selected based on reproducible amplification and absence of aspecific bands.

## 4. Conclusions

This set of microsatellite loci is the first for a non-endangered Australian alpine animal. These loci provide us with the opportunity to gather baseline data on an abundant and ecologically important species in this region. This is significant because the Australian alpine ecosystem is highly threatened by climate change.

## Figures and Tables

**Table 1:** Primer sequences, repeat motifs, size ranges Forward primers were tagged with a 5′M13 universal sequence (5′-TGTAAAACGACGGCCAGT-3′), but size ranges shown excluded this portion of the sequence and Genbank accession numbers for *K. trisits* microsatellite loci.

Locus	Primer Sequences (5′–3′)	Repeat Motif	Size Range (bp)	Genbank Accession No.
Ktr29	F: TAACTGGCAGACAGGCACAC	(AC)_19_atg(CA)_7_	176–202	HQ316183
	R: AGGCCTTAGAGCCCAGAGAC			
Ktr30	F: GCAAATTTGGCGTCTTCATC	(AC)_12_	144–206	HQ316184
	R: GTCATAAGGGCGGATACTGG			
Ktr58	F: CAGTTACATACAGCAACATGTCTAGG	(GA)_5_a(AG)_19_	242–314	HQ316185
	R: AGCTTCAAGCAAGACTGAGATG			
Ktr60	F: TCACCGGTATGGCTCTTAGG	(TG)_18_	218–254	HQ316186
	R: TTCGGCGTGAAGACGTAAC			
Ktr73	F: GGGAATTTGAATCTGTGATCG	(TG)_10_ta(TG)_8_	242–250	JX843414
	R: CACGACGTTGTAAAACGAC			
Ktr76	F: GTAACTGAGCACCTGGCACA	(TG)_12_	234–238	HQ316187
	R: GCAGACCGAGGATGAGAAAG			
Ktr82	F: TCCAGTCTTGTCAGGAAATACG	(TG)_19_	196–218	HQ316188
	R: TGCTGCAATCTCATCAATGG			
Ktr88	F: ACATGCCGGTGCCATTTAC	(AC)_8_aa(AC)_20_	176–216	HQ316189
	R: CAATGATTTCCGTGGATACG			

**Table 2:** Characteristics of microsatellite loci for *Kosciuscola tristis*.

Locus	Dye	Pf (μM)	Pr (μM)	MgCl_2_ (μM)	*N*	*N*_test_	*N*_A_	*H*_O_	*H*_E_	*p*	Null Allele Freq.
Ktr29	f	0.02	0.10	2.0	26	26	15	0.692	0.907	0.32	0.10
Ktr30	p	0.03	0.10	2.5	24	26	16	0.333	0.907	<0.01	0.30
Ktr58	p	0.03	0.10	2.5	24	26	4	0.583	0.497	0.82	0.00
Ktr60	n	0.03	0.10	2.5	25	26	17	0.760	0.874	1.00	0.03
Ktr73	f	0.02	0.10	2.5	25	30	3	0.160	0.215	0.42	0.07
Ktr76	v	0.02	0.10	2.5	23	26	20	0.435	0.910	<0.01	0.25
Ktr82	n	0.02	0.10	2.5	25	26	12	0.200	0.852	<0.01	0.35
Ktr88	v	0.03	0.10	2.5	23	26	12	0.565	0.868	0.03	0.15

Dye: dye on M13 tag used for each locus (p: pet, f: fam6, v: vic, n: ned), Pf: forward primer concentration; Pr: reverse primer concentration, MgCl_2_: concentration; *N*: number of grasshoppers successfully genotyped; *N*_test_: number of samples for which amplification was attempted, *N*_A_: number of alleles; *H*_O_: observed heterozygosity; *H*_E_: expected heterozygosity; *p*: probability of deviation from Hardy-Weinberg equilibrium, Null Allele Freq.: null allele frequencies computed with the method of [14] using FreeNA [12].
